# Niche separation and weak interactions in the high tidal zone of saltmarsh‐mangrove mixing communities

**DOI:** 10.1002/ece3.7263

**Published:** 2021-03-25

**Authors:** Patrick Ndayambaje, Lili Wei, Tingfeng Zhang, Yuhong Li, Lin Liu, Xu Huang, Chaoxiang Liu

**Affiliations:** ^1^ Key Laboratory of Urban Pollutant Conversion Institute of Urban Environment Chinese Academy of Sciences Xiamen China; ^2^ University of Chinese Academy of Sciences Beijing China; ^3^ Huaqiao University Xiamen China

**Keywords:** adult species interaction, coastal wetland species, coexistence, niche differentiation, niche width, plant functional traits

## Abstract

1. Saltmarsh‐mangrove ecotones occur at the boundary of the natural geographic distribution of mangroves and salt marshes. Climate warming and species invasion can also drive the formation of saltmarsh‐mangrove mixing communities. How these coastal species live together in a “new” mixed community is important in predicting the dynamic of saltmarsh‐mangrove ecosystems as affected by ongoing climate change or human activities. To date, the understanding of species interactions has been rare on adult species in these ecotones.
2. Two typical coastal wetlands were selected as cases to understand how mangrove and saltmarsh species living together in the ecotones. The leaves of seven species were sampled from these coastal wetlands based on their distribution patterns (living alone or coexisting) in the high tidal zone, and seven commonly used functional traits of these species were analyzed.
3. We found niche separation between saltmarsh and mangrove species, which is probably due to the different adaptive strategies they adopted to deal with intertidal environments.
4. Weak interactions between coexisting species were dominated in the high tidal zone of the two saltmarsh‐mangrove communities, which could be driven by both niche differentiation and neutral theory.
5. Synthesis. Our field study implies a potential opportunity to establish a multispecies community in the high tidal zone of saltmarsh‐mangrove ecotones, where the sediment was characterized by low salinity and high nitrogen.

## Graphical abstract

We found niche separation between saltmarsh and mangrove species and weak interaction between coexisting species in the high tidal zone of the saltmarsh‐mangrove mixing communities.

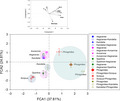

## 1 INTRODUCTION

**FIGURE 1:**
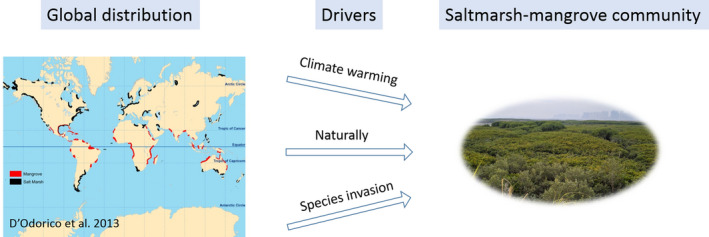
The distribution and formation of saltmarsh‐mangrove community

Mangroves and saltmarshes have been considered as highly cherished ecosystems in providing many important ecological functions and services (Armitage et al., 2015; Barbier et al., 2011; Kelleway et al., 2017; Yando et al., 2016). But in general, they have distinct latitudinal ranges with mangroves confined to tropical or subtropical regions and replaced by saltmarshes in higher latitudes (D'odorico et al., 2013; Pickens et al., 2019). Extreme air temperature is a crucial factor to limit the poleward expansion of mangroves (Osland et al., 2017); therefore, near the latitudinal limit of mangroves, saltmarsh‐mangrove mixing community emerged naturally. In addition, the mixing community could also be caused by a consequence of human activities (Figure 1). For example, climate warming has caused the formation of the saltmarsh‐mangrove mixed community on the east coast of Florida (Cavanaugh et al., 2014), the northern Gulf of Mexico coast, USA (Guo et al., 2013), the south‐eastern Australia (Kelleway et al., 2016), and many other coasts worldwide (Saintilan et al., 2014). In China, species invasion has resulted in the coexisting of mangrove and saltmarsh species, such as in the coasts in Fujian, China (Zhang et al., 2012). Due to the increasing human disturbances, mangrove and saltmarsh species could have more opportunities to live together. How they influence each other and whether they can live together stably are still not fully understood due to the lack of adequate knowledge about the interactions between these coastal species.

An ecological niche characterizes the position of a species within an ecosystem, comprising both the habitat requirements and the functional role of a species (Polechová & Storch, 2008). The natural distribution of mangroves and saltmarshes reflects a distinct niche partitioning related to temperature limit (Saintilan et al., 2014; Schaeffer‐Novelli et al., 2016), but when they are living under the same climate conditions, whether the adult plants maintain such niche partitioning has remained unclear, because the previous studies focused mainly on the species interactions during their early growing stages (e.g., Lewis, 2005; Li et al., 2014; McKee & Rooth, 2008). Based on the fact that both mangrove and saltmarsh species adapt to coastal environments such as saline and flooding (Boorman, 2003), we expected that they would show niche overlap in the mangrove‐saltmarsh ecotones.

The study of the interaction in mangrove‐saltmarsh ecotones has paid particular attention to *Spartina alterniflora* and *Avicennia germinans*. *Spartina alterniflora* is among the most widely distributed saltmarsh species and has invaded into mangrove communities in many countries (Zheng et al., 2018). *Avicennia* is the most cold‐tolerant mangrove genus, and their encroachment into saltmarshes occurred in Australia and USA coastal wetlands as the consequence of climate change (Kelleway et al., 2017; Saintilan et al., 2019). These previous studies indicate that the interaction between *S. alterniflora* and *A. germinans* varies with plant growth stages. *Spartina alterniflora* can facilitate mangrove recruitment by trapping floating propagules (Lewis III, 2005). By contrast, the recruitment of *S. alterniflora* is commonly suppressed by mangroves (Li et al., 2014). Once mangrove seedlings are successfully established within stands of marsh vegetation, competitive interactions can be developed due to competition for light (Howard et al., 2015; Pickens et al., 2019). For example, if their height exceeds that of co‐occurring marsh species, mangrove seedlings gain a competitive advantage by inducing light limitation for marsh species (Patterson et al., 1997). While if *S. alterniflora* has greater aboveground biomass than coexisting *A. germinans* seedling, such as in Louisiana, USA, *S. alterniflora* suppresses the growth of *A. germinans* seedling (McKee & Rooth, 2008).

So far, only a few studies have explored the interaction among the coastal wetland species other than *S. alterniflora* and *A. germinans*. Even so, these studies obtained controversial results. For instance, McKee et al. (2007) found marsh species *Sesuvium portulacastrum* and *Distichlis spicata* facilitated the establishment of *Rhizophora mangle* by increasing soil redox potential and decreasing soil temperature and salinity. But Howard et al. (2015) found that *D. spicata* and another saltmarsh species *Eleocharis cellulosa* impacted negatively on the growth of mangrove seedlings. In addition, the interactions of these species also changed throughout a species life history (Pickens et al., 2019). For example, the effect of salt marsh vegetation on mangrove species *A. germinans* switched from negative to neutral as *A. germinans* growing from seedling to juvenile trees (Guo et al., 2013).

Plant–plant interaction is typically indicated by plant growth or aboveground biomass. However, plant growth represents the net balance of the interaction, which is impossible to distinguish competition and facilitation simultaneously (Armas, 2004). Because traits offer a taxon‐independent comparison between species, trait‐based approaches have been increasingly recognized to be a better way of understanding community assembly and species coexistence (Adler et al., 2013; Cadotte et al., 2015). For example, Kraft et al. (2015) using 11 functional traits quantified average fitness and stabilizing niche differences between 102 plant species pairs and confirmed that individual traits were correlated with fitness differences, but stabilizing niche differences can only explain by the analysis of combination traits.

Based on the stress‐gradient hypothesis, species interactions are competitive in benign and facilitative in harsh conditions (Maestre et al., 2009; Qi et al., 2018). Generally, species living in similar environments are competing for the same resources (Connell, 1983; Tarjuelo et al., 2017). To avoid competition, most species will have different traits from each other. Therefore, plant species are likely more diverse in resource‐acquisition traits when they coexist than when live alone. The saltmarsh‐mangrove ecotones in China often include *Spartina anglica* and *S. alterniflora* which were introduced in 1963 and 1979, respectively, and have successfully reproduced on the Chinese coasts (Zuo et al., 2012). Evidence has been increasingly presented that *Spartina spp*. out‐competed almost all the native plants such as *Phragmites australis* and *Scirpus mariqueter* (An et al., 2007). Therefore, we expected more divergent traits between coastal species to occur when they coexisting than when they living alone, and facilitative interaction is more likely between native species while competition between invasive and native species.

The present study aimed to gain a trait‐based understanding of species coexistence of coastal wetland species in the saltmarsh‐mangrove ecotones by testing two hypotheses: (a) niche overlap occurs between saltmarsh and mangrove species; (b) facilitative interaction is dominated in the saltmarsh‐mangrove communities. We analyzed the most common mangrove and saltmarsh species to examine the niche distribution by trait combination analysis and pairwise interactions by comparing the leaf functional traits of the coexisting species pairs.

## 2 MATERIALS AND METHODS

### 2.1 Study sites and sampling

**FIGURE 2:**
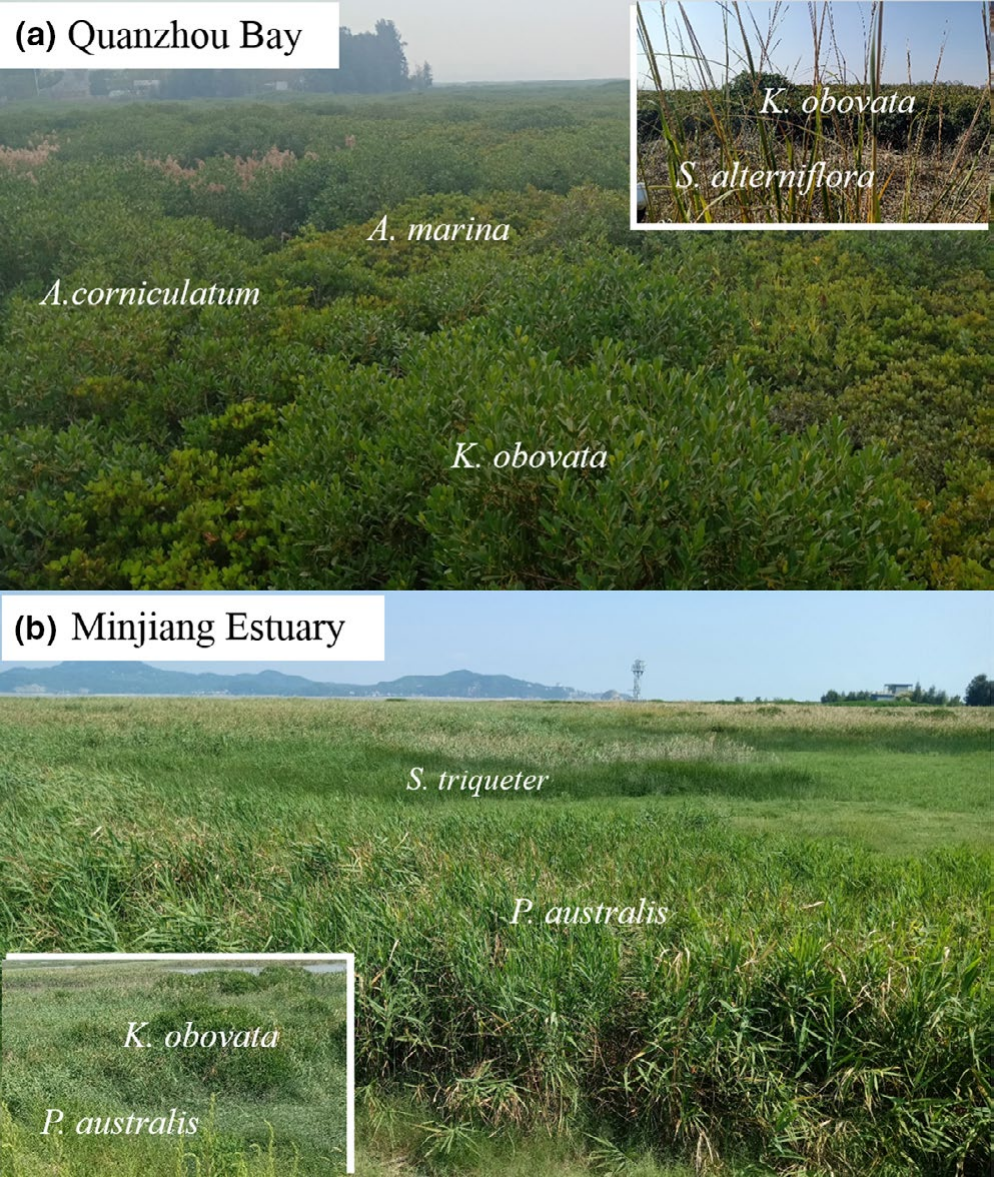
The species collected from (a) Quanzhou Bay Mangrove Reserve and (b) Minjiang Estuary wetlands

In China, the range limitation of the geographic distribution of mangroves is located in the northern area of Fujian Province. Therefore, we carried out our fieldwork in two coastal wetlands in north Fujian. Quanzhou Bay Mangrove Reserve (24°57'24''N–118°41'25''E) has a total area of 7,130 ha. The annual mean temperature is 20.4°C, and the average annual precipitation is 1,095.4 mm. The prevalent climate of this region is an oceanic monsoon climate, characterized by a warm and wet winter and a hot and rainy summer (Lu et al., 2018). This site was dominated by three common mangrove species *Avicennia marina*, *Aegiceras corniculatum*, *Kandelia obovate,* and one invaded saltmarsh species *Spartina alternifora.* The other site is located in Minjiang Estuary wetlands in Fuzhou (26°01'46.2''N–119°37'31.9''E), a tidal beach wetland affected by salt intrusion, located at the estuary of the Minjiang River. The average annual temperature is 19.3°C, and the average annual rainfall is 1,346 mm with about 153 rainy days (Li et al., 2018). This site is dominated by two common saltmarsh species *Scirpus triqueter* and *Phragmite australis* and emerged occasionally with one mangrove species *Kandelia obovata* (Zhou et al., 2006; Figure 2b).

We need to find the same species living alone (apart from the neighbors at least 100 m away) and coexisting with the neighbors (mixed). Interestingly, the species we found that meet our requirement distributed mostly in the high tidal zone. Therefore, we restricted our sampling in the high tidal zone to diminish the difference in sediment conditions. We found seven species in these two sites, with three species pairs from Quanzhou Bay Mangrove Reserve and two pairs from Minjiang Estuary wetland. These species pairs were *A. marina* versus *A. corniculatum, K. obovata* versus *A. corniculatum, S. alterniflora* versus *A. corniculatum, S. triqueter* versus *P. australis,* and *K. obovata* versus *P. australis* (Figure 2a). But in Minjiang Estuary wetland, we did not find *K. obovata* living alone, only a small patch of *K. obovate* has emerged within the *P. australis* community. For each species pair, there were three subplots with an interval of ca. 10 m in each plot. These subplots were treated as replicates because we only found one plot for each pair in our sites.

The full opened and healthy green leaves were selected, and, in each subplot, 30 leaves in total were collected from 15 individuals randomly. Three sediment samples (top 15 cm) for each subplot were collected on the same days with leaf sampling. The three sediments of each subplot were then mixed evenly for chemical analysis. All the samples were packed in sealed bags and brought to the laboratory immediately after field collection.

### 2.2 Trait analysis

Seven functional traits were analyzed to represent important features of plant ecological strategies, including leaf carbon (C), leaf nitrogen (N), leaf phosphorus (P), specific leaf area (SLA), leaf dry matter content (LDMC), leaf area, and leaf biomass. For *S. triqueter*, we measured the traits of stem instead of leaf traits, including stem volume, stem‐specific density (SSD), and twig dry matter content (TDMC), because this species is nearly leafless. Leaf C is composed of defensive and storage compounds, representing partly plant defensive or tolerance capability (Imaji & Seiwa 2010). Leaf nutrients and SLA are the integral components of the worldwide leaf economic spectrum, indicating a trade‐off between an investment in leaf surface area to capture light for photosynthesis and investment in constructing more protective tissues (Liu et al., 2018). High SLA values indicate thinner or less dense leaf tissue, associated with shorter leaf life span and higher metabolic rates per unit mass, while low SLA values occur in evergreen taxa, with lower instantaneous metabolic rates, but enhanced nutrient and water use efficiency (Reich, 2014). LDMC, the ratio of leaf dry mass to its fresh mass, is an indicator of many critical aspects of plant growth and survival (Ali et al., 2019) and broadly utilized as an indicator of a species resource use strategy (Vaieretti et al., 2007). Leaf area and leaf biomass have a significant impact on the exchange of energy, light interception, C cycling, and plant growth (Nowak, 1996; Weraduwage et al., 2015). Leaf area is important for leaf energy, water balance, and tolerance to environmental stress, with smaller leaves generally observed in stressful conditions (Satdichanh et al., 2015).

We measured or calculated these plant functional traits as follows. The leaf area was measured with image analysis software (Image P); then, leaves were dried for 48 hr at 65°C, weighed for determination of SLA, defined as the area of one side of a fresh leaf divided by its oven‐dried mass, and expressed in cm^2^/g (Cardinale et al., 2012). The leaf biomass was considered as leaf dry weight. The leaf C and leaf N were determined with the elemental analyzer (Vario MAX, Vario MACRO, Germany Elementar) after the leaf samples were dried in the oven for 72 hr at 65°C, ground, and then sieved through 60 mesh sieves. Leaf P was analyzed with ICP‐OES (Inductively Coupled Plasma Optical Emission Spectrometer, Optimal 7000DV, PerkinElmer USA). The measurement of stem volume, SST, and TDMC followed the protocol by Cornelissen et al. (2003).

### 2.3 Analysis of sediment properties

Total C, total sulfur (S), and total N were determined by the elemental analyzer (Vario MAX, Vario MACRO, Germany Elementar) after the sediment samples were ground through 60 mesh sieves, weighed 200 mg in white ceramic tubes. For total P, digestion was performed with nitric and perchloric acid; quantification was performed with ICP‐OES (Inductively Coupled Plasma Optical Emission Spectrometer, Optimal 7000DV, PerkinElmer USA). Active pH was determined using deionized water (1:2). Then, EC (electricity conductance) was determined using deionized water (1:5) with electrical conductivity meter and then converted to commonly used salinity unit ‰.

### 2.4 Calculation

To quantitatively estimate the interaction between coexisting species, we calculated three commonly used indexes: RNE (Markham, Chanway, & soil, 1996), lnRR (Hedges et al., 1999), and RII (Armas et al., 2004).

(1) $$\text{RNE}=\frac{P_{s}‐P_{w}}{X}$$

(2) $$\text{lnRR}\;=\ln\left(\frac{P_{w}}{P_{s}}\right)$$

(3) $$\text{RII}=\frac{P_{w}‐P_{s}}{P_{w}+P_{s}}$$

Where, *P_w_* and *P_s_* are the performance of plants with and without neighbors, respectively. RNE and RII range from −1 to 1, while lnRR has no limits (Armas et al., 2004). In RNE, *X* = *P_s_* if *P_s_* > *P_w_*, and *X* = *P_w_* if *P_w_* > *P_s_*, and negative values indicate facilitation and positive values indicate competition between species. In contrast, negative and positive values indicate competition and facilitation, respectively, in RNE and RII.

### 2.5 Statistical analysis

We used IBM SPSS Statistics 23 for all statistical analyses, and statistical significance was set at a *p*‐value equal to <.05 or .01. All data were checked for the normal distribution, and homogeneity of variances before the statistical analysis was carried out. The assessment of the differences in leaf functional traits between the coexisting species and living alone species was carried out by an independent *t* test. The difference in traits among mangrove species and saltmarsh species was analyzed by one‐way ANOVA. To view the niche distribution of all the test species, PCA analysis was carried out and composed of all the traits, in which, stem functional traits of *S. Triqueter,* SSD, and TDMC merged into SLA and LDMC, respectively. To verify whether trait variation is divergent when species coexisting compared with when they living alone, Euclidean distance analysis of each trait was conducted separately through correlate‐distance analysis process in SPSS and then compared trait distance between species when coexisting and when living alone by independent *t* test.

## 3 RESULTS

### 3.1 Niche distribution indicated by trait space

**FIGURE 3:**
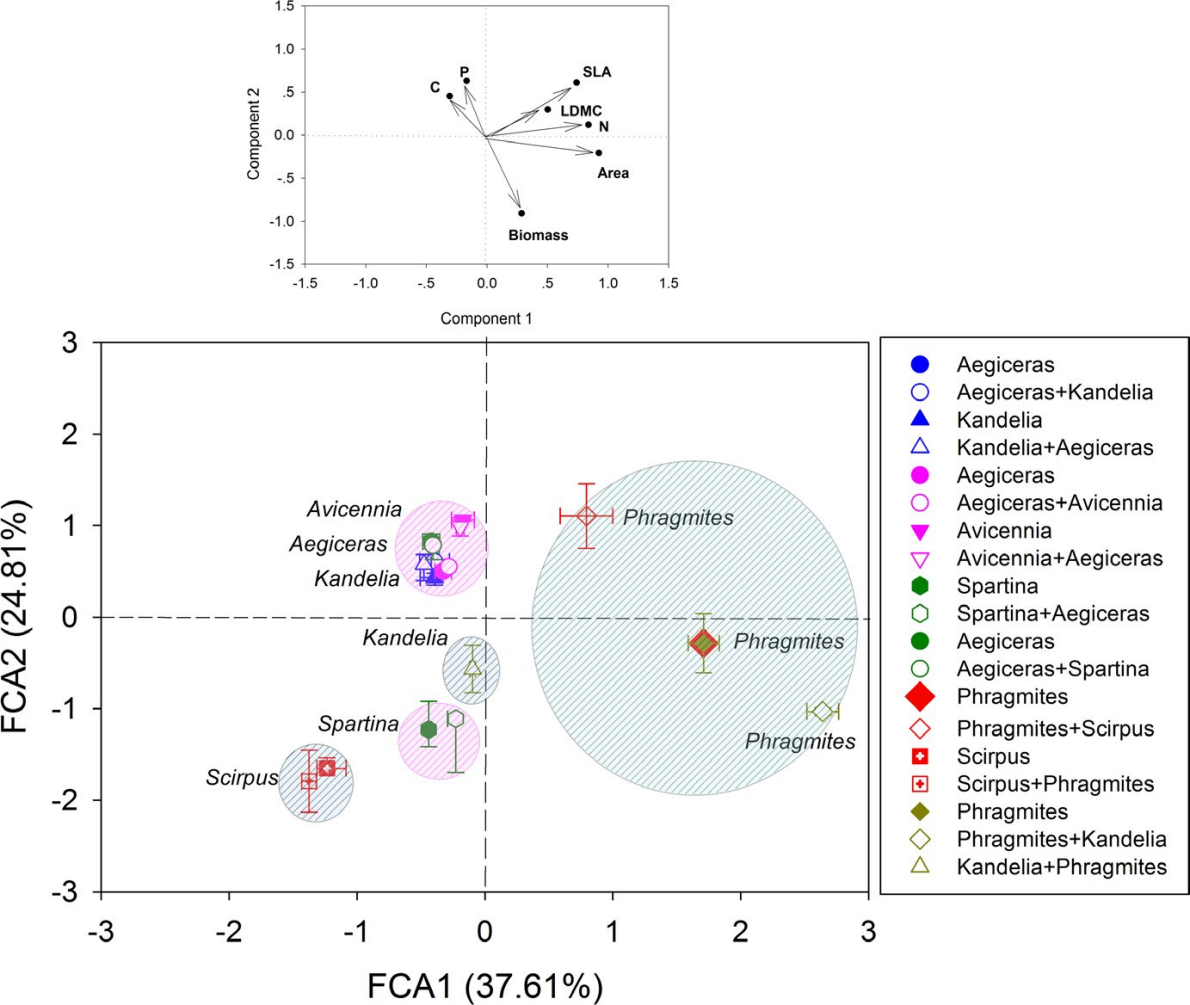
The trait space constructed by common coastal wetland species (close and open symbols represent the niche of the species when living alone and when living with other species, respectively; the same color indicates the coexisting species pair; violet and sienna circles indicate the two wetlands in Quanzhou Bay and Minjiang Estuary, respectively)

PCA analysis separated the traits into two main components. Component 1 is composed of leaf area, leaf N, SLA, and LDMC. Component 2 is composed of leaf biomass, leaf P, and SLA. PCA based trait space showed that the three mangrove species in Quanzhou wetland had similar niche ranges and which were not significantly altered by whether they lived with neighbors, but their niche ranges distinctly differed from the niche of *S. alterniflora* (Figure 3). This is likely related to the differences in leaf biomass and SLA (component 2). In the Minjiang Estuary, mangrove species *K. obovata* had a different niche range from the coexisting saltmarsh species *P. australis* (Figure 3). It is likely due to the differences in leaf area, SLA, and leaf *N* (component 1).

### 3.2 Trait differences between saltmarsh and mangrove species

**FIGURE 4:**
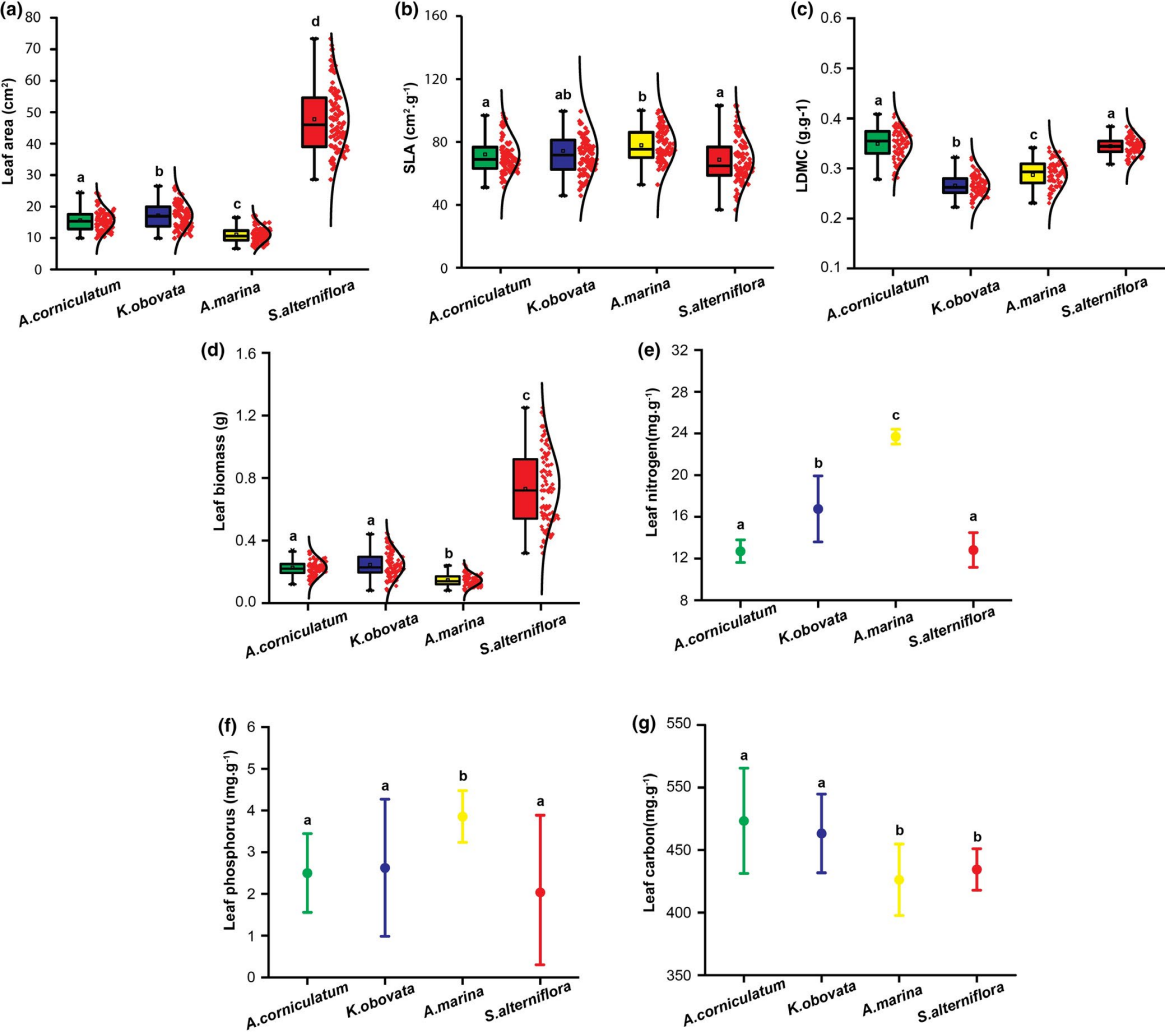
Trait comparison between saltmarsh and mangrove species in Quanzhou Bay wetlands. Leaf area (a), SLA (b), LDMC (c), Leaf biomass (d), Leaf *N* (e), Leaf P (f), and Leaf C (g). The following colors represent green (*A. corniculatum*), blue (*A. marina*), yellow (*K. obovata*), and red (*S. alterniflora*). The interval plot (NPC) and box plots show standards boxes summarize median, first, and third quartile, while whiskers represent value range (minimum and maximum). The red points and the curved line represent the distribution of the data. The symbols with different letters denote significantly different from each other at *p* < .05, while the same letters denote no significant difference (*p* > .05)

**FIGURE 5:**
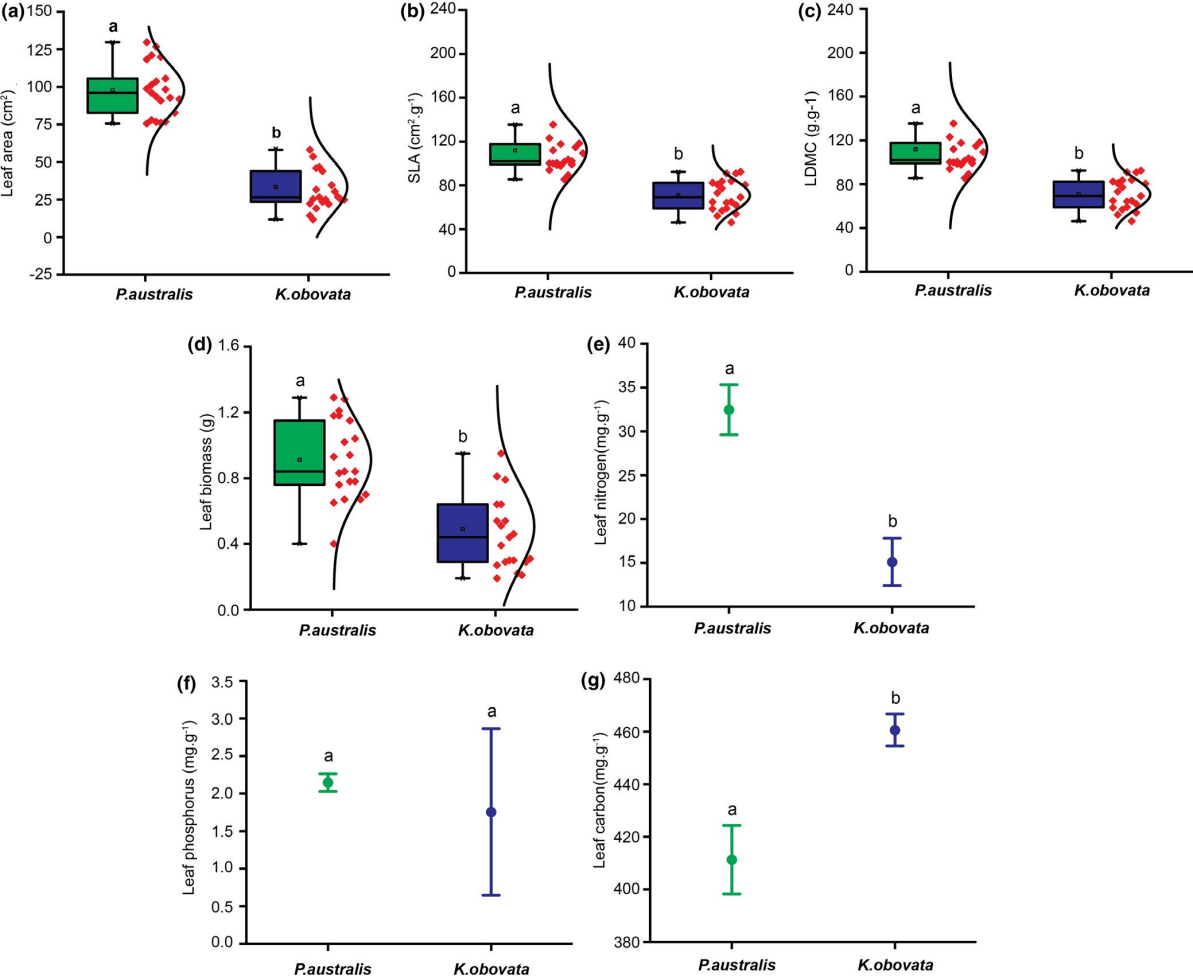
Trait comparison between saltmarsh and mangrove species in Minjiang Estuary wetlands. Leaf area (a), SLA (b), LDMC (c), Leaf biomass (d), Leaf *N* (e), Leaf P (f), and Leaf C (g). Green and blue colors represent *P. australis* and *S. alterniflora,* respectively. The box plots show standards boxes summarize median, first, and third quartile, while whiskers represent value range (minimum and maximum). The red points and the curved line represent the distribution of the data. The symbols with different letters denote significantly different from each other at *p* < .05, while the same letters denote no significant difference (*p* > .05)

In Quanzhou Bay, the SLA and leaf biomass of mangrove species were substantially different from those of *S. alterniflora* (Figure 4b,d). In Minjiang Estuary, mangrove species *K. obovata* and saltmarsh species *P. australis* had distinct differences in most traits except leaf P (Figure 5). These were consistent with the result of the PCA analysis above.

### 3.3 Trait variations when coexisting

**FIGURE 6:**
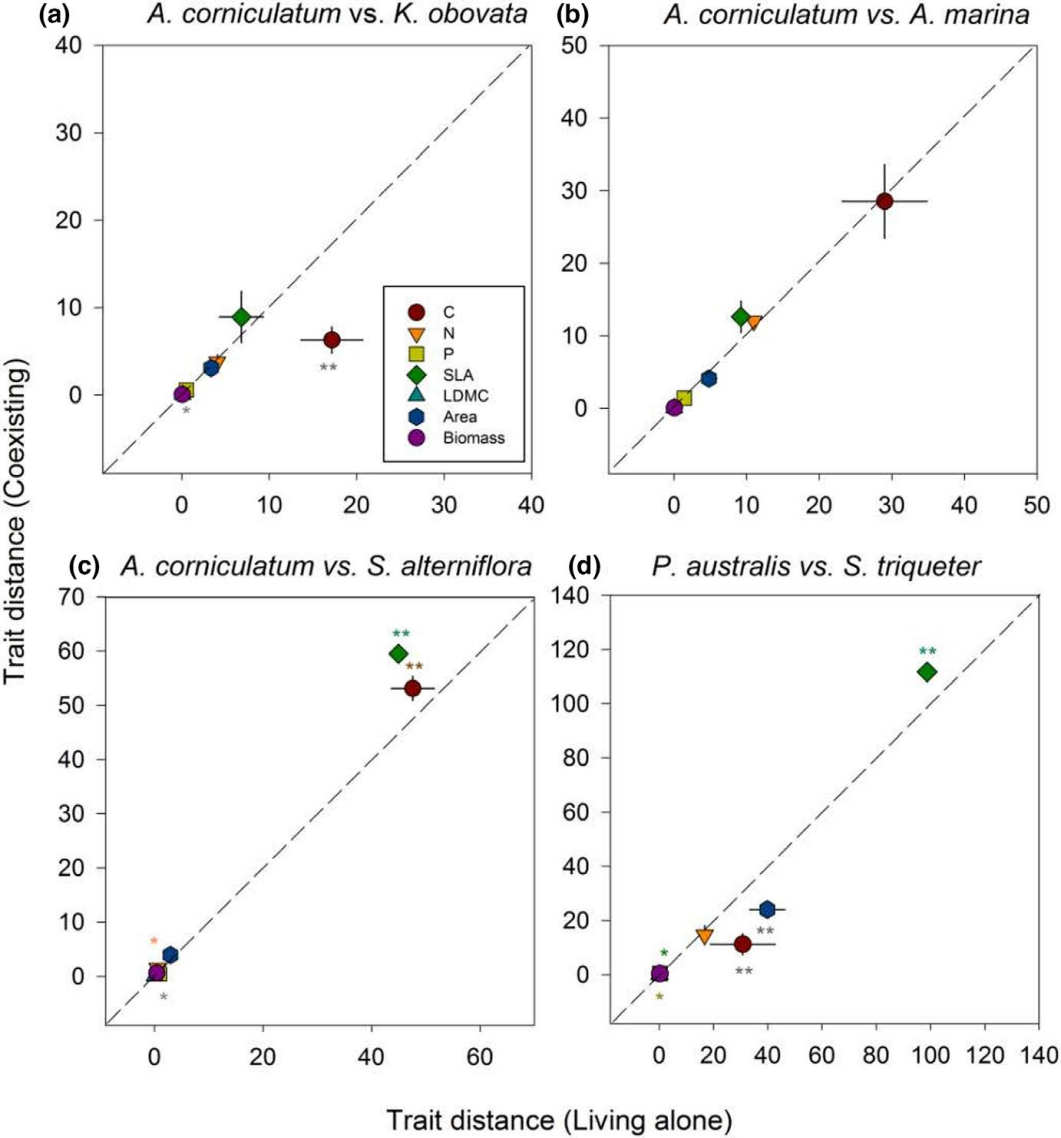
Trait distance when species coexisting and living alone. The asterisk * and ** indicate significant difference at *p* < .05 and *p* < .01, respectively. The colored asterisk represents divergent variation, and the dark color represents trait convergent. The dash line denotes the threshold between divergence and convergence in trait variation. Only when the data within the top‐left space, it means species coexistence leading to divergent variation of a given trait

In general, there was no trait divergence of mangrove species when they coexisting compared with when they living alone (Figure 6a, b), the divergent variations were found only in SLA and leaf C of the pair *A. corniculatum* versus *S. alterniflora* (Figure 6c) and in SLA of *P. australis* versus *S. triqueter* (Figure 6d).

### 3.4 Species interactions

**FIGURE 7:**
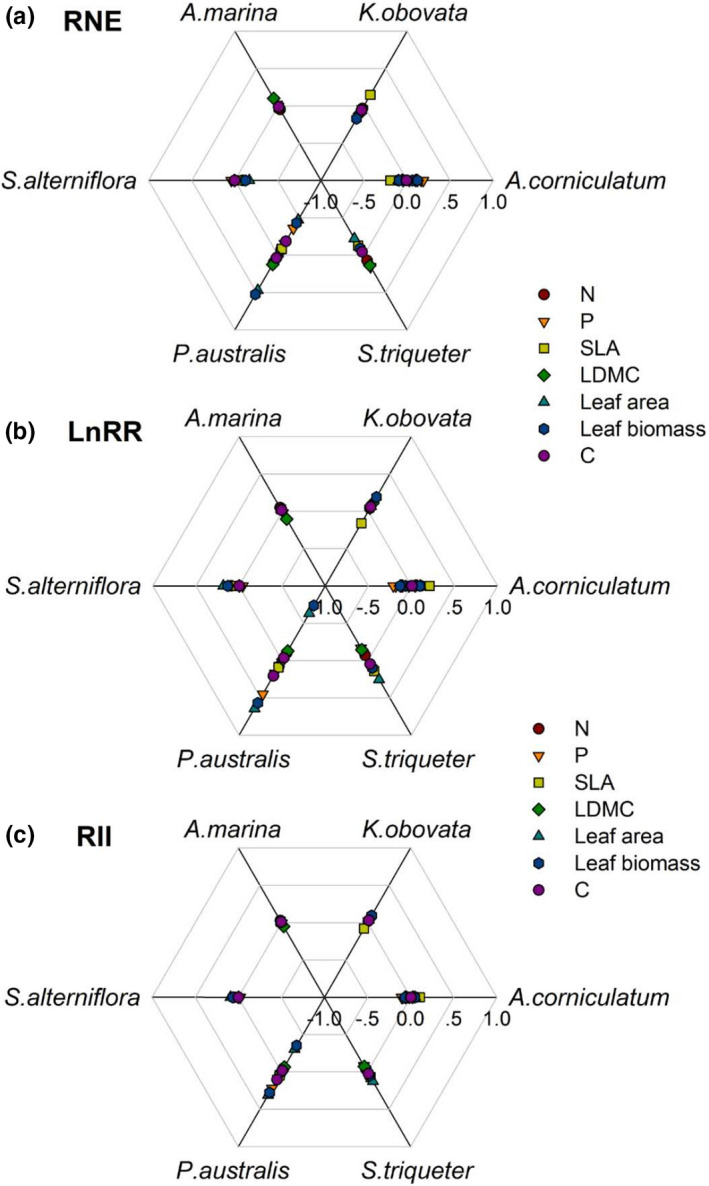
The interactions between species of each trait based on the index of RNE (a), LnRR (b) and RII (c)

In general, the positive or negative interactions between most coexisting species were weak (close to zero; Figure 7), especially the results indicated by the RII index (Figure 7c). Only *P. australis* showed large negative or positive values indicated by leaf biomass and leaf area (Figure 7).

### 3.5 Sediment conditions

The two wetlands showed significant differences in sediment conditions (*p* < .05), but most parameters were not different within each site (Table 1).

**TABLE 1:** The sediment conditions of each plot in the two wetlands sites (Mean ± *SE*)

Plot	N (mg/g)	P (mg/g)	S (mg/g)	pH	Salinity (‰)
Quanzhou Bay					
*A.corniculatum + K.obovata*	1.31 ± 0.36	0.21 ± 0.03*	2.16 ± 2.67	7.06 ± 0.4	3.89 ± 0.79**
*A.corniculatum*	1.71 ± 0.21	0.24 ± 0.05	2.01 ± 1.38	7.27 ± 0.1	4.39 ± 0.11
*K.obovata*	1.53 ± 0.48	0.38 ± 0.07*	1.02 ± 0.25	7.00 ± 0.2	6.49 ± 0.27**
*A.corniculatum + A.marina*	1.48 ± 0.17	0.30 ± 0.00	2.11 ± 1.50	7.18 ± 0.2	4.53 ± 0.11
*A.corniculatum*	1.76 ± 0.06	0.24 ± 0.04	3.91 ± 1.43	7.01 ± 0.1	4.88 ± 0.47
*A.marina*	1.67 ± 0.07	0.32 ± 0.03	3.36 ± 0.21	7.11 ± 0.1	4.46 ± 0.3
*A.corniculatum + S.alterniflora*	1.44 ± 0.29	0.26 ± 0.08	1.76 ± 1.53	7.67 ± 0.1	3.02 ± 0.13**
*A.corniculatum*	1.71 ± 0.21	0.24 ± 0.05	2.01 ± 1.38	7.06 ± 0.4	4.39 ± 0.11**
*S. alterniflora*	1.60 ± 0.27	0.25 ± 0.01	2.27 ± 1.36	7.48 ± 0.3	2.3 ± 0.47
Minjiang Estuary					
*P. australis + S. triqueter*	2.25 ± 0.30	0.25 ± 0.07	0.28 ± 0.10	6.24 ± 0.3	0.7 ± 0.07*
*P. australis*	2.32 ± 0.24	0.32 ± 0.02	0.14 ± 0.04	6.12 ± 0.2	0.55 ± 0.05*
*S. triqueter*	3.10 ± 0.52	0.39 ± 0.12	0.59 ± 0.27	5.92 ± 0.2	0.98 ± 0.35
*P. australis + K. obovata*	2.70 ± 0.23	0.83 ± 0.07**	0.30 ± 0.04**	6.27 ± 0.2	1.39 ± 0.26**
*P. australis*	2.32 ± 0.24	0.32 ± 0.02**	0.14 ± 0.04**	6.12 ± 0.2	0.55 ± 0.05**

Note

The significant differences in sediment profiles between the plots when species living alone and together were denoted by *p < .05 and **p < .01.

## 4 DISCUSSION

Generally, niche separation was found between mangrove and saltmarsh species, and weak interactions between coexisting species were dominated in the high tidal zone of the two saltmarsh‐mangrove ecotones. The niche separation was likely due to the different adaptive strategies between saltmarsh and mangrove species, while the weak interactions could be due to the niche differentiation and neutral theory.

### 4.1 Niche separation between mangrove and saltmarsh species

A recent study has confirmed that trait combination analysis is a better way to indicate niche differences than single functional traits (Kraft et al., 2015). Our combination trait analysis integrated multiple niche dimensions. The traits we used in this study fall into three well‐established trait dimensions: leaf economic traits (leaf N, P, SLA, and LDMC) that indicating resource use strategy (Wright et al., 2004), leaf C that characterizing plant defense or tolerance ability (Lovelock et al., 2016; McKee, 1995; Parida & Jha, 2010; Reef et al., 2010), and leaf area and leaf biomass that representing resource acquisition and plant growth (Nowak, 1996; Weraduwage et al., 2015). Therefore, our trait integrative analysis indicated multidimensional niche differences. This has an advantage in predictive of coexistence mechanism and community assembly processes compared with the single trait such as aboveground biomass or productivity that previously commonly used.

The trait space built by PCA analysis in our study provided a clear picture of the niche separation between saltmarsh and mangrove species (Figure 3). Further, the trait comparison between the two types of coastal vegetation was consistent with the results from PCA analysis (Figures 4 and 5). Specifically, saltmarsh species had a greater growth trait; in contrast, mangrove species had higher defensive traits, indicating that mangrove and saltmarsh species adopted different strategies in adaptation to intertidal environments. Mangrove species commonly form conservative strategies such as slow‐growing, succulence but high in leaf C and phenolic compounds that confer its strong ability in tolerance to stressful environments such as salinity, aridity, inundation, extremes of temperature, and excessive solar radiation (Lovelock et al., 2016; McKee, 1995; Parida & Jha, 2010; Reef et al., 2010). In contrast, *S. alterniflora* and *P. australis* respond to stressful environments by enhancing their ability in resource use, such as greater leaf biomass and larger leaf area, to obtain resources quickly during the growth period (the aboveground tissues die in winter).

Collectively, niche separation occurred in our saltmarsh‐mangrove communities (Figure 3). Our study provides empirical evidence in the saltmarsh‐mangrove communities, improved our understanding of the niche differences in multidimensional nature. Despite a similar phenomenon was also observed in animal communities such as at the *Sauropod omorph*–sauropod boundary (McPhee et al., 2015), we have not found similar observations reported in plant communities at the geographic boundary.

### 4.2 Weak interactions dominated in the high tidal zone

Coastal wetlands are usually under highly stressful environmental conditions, such as saline, anaerobic, high intensity of sunlight, and low nutrients (Lovelock et al., 2016; McKee, 1995; Parida & Jha, 2010; Reef et al., 2010); thus, positive interactions (mutualism and facilitation) are supposed to be one of the best ways to mitigate environmental stresses based on stress‐gradient hypothesis (He et al., 2013; Renzi et al., 2019). However, in our study, no divergence in most traits and low values of interaction indexes between most coexisting species indicated that weak interactions dominated in our sites. Specifically, the weak interactions were found between the coexisting pairs of *S. alterniflora* versus *A. corniculatum*, *A. marina* versus *A. corniculatum*, and *K. obovata* versus *A. corniculatum*.

The weak interaction between *A. corniculatum* and *S. alterniflora*, against the general view, which states that *S. alterniflora* outcompetes native species (Ju et al., 2017), although both the competition and facilitation of marsh species on mangrove establishment have been observed (McKee & Rooth, 2008; Patterson et al., 1997). But in our study, this was consistent with the observation of the niche separation between mangrove and saltmarsh species (Figure 3). The dominated weak interactions between coexisting species were likely related to the low salinity in the sediment of our sites (<4.5‰), where we carried out in the high intertidal zone (Table 1). A study on the seedlings also found that *S. alterniflora* had a weak competitive effect on mangrove seedlings at the oligohaline site (Zhang et al., 2012). Even on a coast in China, the native marsh species exhibited competitive advantages over *S. alterniflora* at low salinity habitat (ca. 7‰), but replaced by *S. alterniflora* with salinity increased (Tang et al., 2014). These studies help to interpret, at least partly, the weak interaction between *A. corniculatum* and *S. alterniflora*.

The weak interaction between *A. marina* versus *A. corniculatum* was also related to their niche separation (Figure 3). Specifically, *A. marina* had lower leaf area, leaf biomass, and leaf C but higher leaf N and P than *A. corniculatum* (Figure 4d–g). Despite it distributed in the low sediment salinity in our sites (Table 1), *A. marina* is an originally highly salt‐tolerant species, and it can use multiple pathways, including salt excretion, salt accumulation, and salt secretion, to deal with high salinity (Parida & Jha, 2010). The lower values in growth traits in *A. marina* are the results of the trade‐off between growth and salt tolerance (Ball & Pidsley, 1995). In addition to salt tolerance, *A. marina* also shows a different response to chilling from *A. corniculatum* (Peng et al. 2015). Our results indicated that *A. marina* has different niches from *A. corniculatum* and showed strong niche conservatism, which is defined as the retention of niche‐related ecological traits over time (Wiens et al., 2010).

Regarding *K. obovata* and *A. corniculatum*, they are the most common mangrove species in China. Particularly in Fujian, large areas of mangroves were composed of these two species. They have similar ecological strategy and leaf trait values, therefore showed overlap in niche range. The weak interaction between them was more likely related to the neutral theory, which states that species with equivalent functions can be distributed in community by stochastic processes (Stokes & Archer, 2010).

The interaction between *P. australis* and *S. triqueter* was not clear. These two species are both clonal perennial plants and adapt to freshwater/brackish and less inundated habitats (Wang et al., 2010). *P. australis* grows well in brackish tidal habitat, but its stem density or growth can be significantly reduced in nontidal freshwater marshes (Meyerson et al., 2000) or higher saline and flooding habitats (Hellings & Gallagher, 1992; Carus et al., 2017). They have found that can coexist stably in communities such as in an island near Shanghai, China (Shi et al., 2007), but the coexisting plots had different sediment conditions from the plots that *P. australis* growing alone in our study (Table 1). Therefore, it is impossible to identify niche separation or neutral theory driving their coexistence based on the current study.

The weak interactions between species in our study were related to both neutral and niche separation, indicating that community assembly is more likely driven by multiple processes, rather than only one process assuming to be sufficient to account for community structure (Stokes & Archer, 2010).

In the natural world, the coastal wetlands are much smaller than the terrestrial forests in terms of the occupation area. Further, the saltmarsh‐mangrove ecotones only distribute at the geographic boundary between these communities. Therefore, it is impossible to find many species and many sites in saltmarsh‐mangrove communities. Even so, our study using integrative analysis of multiple traits provided a relatively clear picture indicating the niche separation between saltmarsh and mangrove species and which were found in both sites. And, the weak interactions were confirmed in three pairs of coexisting species by the quantitative estimation from both the trait distance analysis and the commonly used interaction index. Based on our field‐based observation, further studies with stimulated experiments on more species are the alternative solution to provide more credible evidence.

We here compared only the functional traits of aboveground, therefore cannot exclude the potential belowground competition (Vasquez et al., 2005). The above and belowground could perform differently in response to biotic and abiotic environments (Howard et al., 2018). Despite many studies have found that there is a close relationship between above and below traits, a recent study found that shoot traits were related to root traits only in monocultures, and such relationships were either weak or not detected in mixed communities (De Long et al., 2019). A meta‐analysis concluded that root competition was generally stronger than shoot competition under some conditions, such as at lower nutrient levels (Kiær et al., 2013). Thus, further study on root interaction in saltmarsh‐mangrove ecotones is necessary.

## 5 CONCLUSION

We found niche separation among coastal species and weak interactions between coexisting species dominated in the high tidal zone of the mangrove‐saltmarsh ecotones. The niche separation and the weak interactions we found here implied a potential possibility that building a multispecies community at the high tidal zone of the mangrove‐saltmarsh ecotones. In addition, our multiple uses of plant traits have an obviously advantage in predictive of coexistence mechanism compared with the single trait such as aboveground biomass or productivity that previously commonly used.

## CONFLICT OF INTEREST

The authors have declared that no competing interests exist.

## AUTHOR CONTRIBUTION

**Patrick Ndayambaje:** Data curation (equal); Formal analysis (lead); Investigation (lead); Methodology (lead); Writing‐original draft (lead); Writing‐review & editing (equal). **Lili Wei:** Conceptualization (lead); Data curation (lead); Formal analysis (lead); Funding acquisition (lead); Investigation (lead); Methodology (lead); Project administration (lead); Resources (lead); Supervision (lead); Writing‐review & editing (lead). **Tingfeng Zhang:** Investigation (equal). **Yuhong Li:** Investigation (supporting); Writing‐review & editing (supporting). **Lin Liu:** Writing‐review & editing (supporting). **Xu Huang:** Writing‐review & editing (supporting). **Chaoxiang Liu:** Writing‐review & editing (supporting).

## Data Availability

All relevant data are available via Dryad (https://doi.org/10.5061/dryad.h9w0vt4gz).
